# Shifts in competition outcomes between two *Daphnia* species in response to algal phosphorus content

**DOI:** 10.1007/s00442-025-05669-5

**Published:** 2025-02-07

**Authors:** Irina Feniova, Andrew R. Dzialowski, Anna Bednarska, Tomasz Brzeziński, Natalia Zilitinkevicz, Piotr Dawidowicz

**Affiliations:** 1 Institute of Ecology and Evolution, Russian Academy of Sciences, Moscow, Russia; 2 Department of Integrative Biology, Oklahoma State University, Stillwater, OK USA; 3 Department of Hydrobiology, Institute of Ecology, Faculty of Biology, University of Warsaw, Warsaw, Poland; 4 Water Problems Institute, Russian Academy of Sciences, Moscow, Russia

**Keywords:** Zooplankton, Competition experiments, Computer modeling, Threshold food concentrations, Food quality, Food supply

## Abstract

Competition is one of the key drivers of cladoceran community dynamics. Competitive abilities can be measured as the Threshold Food Concentration (TFC), i.e., concentration of food at which population growth rate equals zero. Species with lower TFC should be superior competitors. However, population TFC can vary with environmental conditions. We hypothesized that algal phosphorus (P) content influences TFC and can alter the outcome of competition between large and small-bodied cladocerans. We conducted competition and life table experiments with single clones of large *Daphnia magna* and small *Daphnia longispina* to assess how algal P content affected their TFC and competitive interactions. We also conducted computer simulations to further explore competition between the small and large species. Our experiments showed that TFC varied with the algal P content. P limitation increased the TFC of both species, but this increase was more pronounced for the smaller *D. longispina*. For this reason, *D. magna* was a superior competitor at low P content, while *D.* *longispina* was superior at high P content. We also found that enhanced food abundance gave an additional advantage to the large-bodied *D. magna* due to higher reproduction potential at high food concentrations.

**Supplementary Information:**

The online version contains supplementary material available at 10.1007/s00442-025-05669-5.

## Introduction

Cladocerans are a keystone group in freshwater ecosystems that control planktonic algae and serve as a valuable resource for higher trophic levels. In the absence of predation, competition is one of the main drivers of cladoceran dynamics (Gliwicz 2003; Bomfim et al. 2023). According to the size-efficiency hypothesis (SEH, Brooks and Dodson 1965), large-bodied cladoceran species are superior competitors over small-bodied species because they are more effective filter feeders and consume a wider range of food particles. The SEH was corroborated by the finding that large-bodied cladoceran species have lower Threshold Food Concentrations (TFC) at which the somatic growth rates equal zero. TFC was assumed as a measure of competitor ability of cladoceran species (Gliwicz 1990, 2003; Gliwicz and Lampert 1990; Achenbach and Lampert 1997; Tessier et al. 2000). However, according to Tilman’s resource competition theory (Tilman 1981, 2004), R* (TFC based on population, not somatic growth rate) is a predictor of competitive ability. The lower R*, the higher competitive ability of the species. Hence, Tilman's R* and TFC based on somatic growth rate data are different predictors of competitive ability. Tilman (1981, 1982) successfully verified the accuracy of the R* assessment of competitive ability in competition experiments with diatoms. Later resource competition theory was affirmed by Rothhaupt (1988) in experiments with rotifers. However, there was no such verification for cladoceran species which differ from algae and rotifers because they exhibit more complex age structure. In addition, there was no verification of the relation between TFC based only on the somatic growth rate and competitive ability. We think that it is questionable that juvenile growth rate can be a robust predictor of the population growth rate which depends on mortality, juvenile development time, and fecundity. We argue that until the direct verification of R*, we cannot assert that resource competition theory predicts the outcome of competition between cladoceran species.

This inconsistency can be the reason why the results of empirical competition experiments between large- and small-bodied cladocerans did not always support the SEH. On the contrary, some studies showed that small-bodied cladoceran species were superior competitors compared to large-bodied species (Lynch 1978; DeMott 1982; Hanski and Ranta 1983; Romanovsky 1984; Romanovsky and Feniova 1985; Dzialowski and O’Brien 2004; Maszczyk et al. 2024). Bengtsson (1987) reviewed hypotheses predicting the outcome of competition between small- and large-bodied species of cladoceran, and found that none had sufficient empirical support to consistently explain relationships between competitive ability and cladoceran body size. For example, the SEH was supported in only 60% of the studies. As a result, Bengtsson (1987) concluded that competitive superiority among cladocerans cannot be attributed only to the body size of competing species. Iwabuchi and Urabe (2012b) also did not find any relationships between body size and competitive superiority in cladocerans. Therefore, the framework to explain the connection between the outcome of competition and cladoceran body size must include a multiple factor approach.

Since 1990s, a great deal of research has suggested that phytoplankton–zooplankton dynamics are controlled at the biochemical level and that zooplankton communities are regulated by the stoichiometric ratios of carbon:phosphorus (C:P) in food resources (DeMott et al. 1998; Sterner and Schulz 1998; Becker and Boersma 2005; Iwabuchi and Urabe 2012a). Nutrients, especially P, are often limiting in lakes. The C:P ratios of phytoplankton can vary greatly, frequently forcing consumers to cope with a diet that does not meet their physiological demands with respect to stoichiometric composition (White 1993). Food quantity and quality, in terms of stoichiometric ratios (C:P) in the phytoplankton biomass, can therefore act in combination to influence outcomes of competition between cladocerans. In support, Iwabuchi and Urabe (2012b) indicated that the TFC is not a fixed characteristic of a population, and can be modified by food quality.

Large- and small-bodied species differ in their stoichiometric composition (Andersen and Hessen 1991). Small-bodied species have been shown to have a lower P content than large species (Andersen and Hessen 1991; Bergström et al. 2015). The growth rate hypothesis (GRH; Elser et al. 1996, 2000) predicts that large-bodied species require more P mainly used to construct ribosomes supporting their rapid somatic growth (Sterner and Hessen 1994; Elser et al. 1996). Therefore, small-bodied cladocerans are predicted to be less sensitive to P limitation than large-bodied taxa.

Iwabuchi and Urabe (2012a, b) showed that TFC based on somatic growth rate in 7 species under low P content (LP) were higher than that in high P content (HP) food conditions, but the magnitude of the changes differed between species. Thus, the rank order of the species in terms of TFC differed when they were fed HP and LP algae. The reason for higher TFC at LP than at HP could be compensatory feeding. When cladocerans are fed with food with low P content, they consume more food to meet their P requirements (Boersma and Kreutzer 2002). As mentioned above, large and small cladoceran species have different requirements for P. For this reason, we assume that changes in R* would differ in small and large species in response to a decrease of the P content in their diet.

As Hart and Bychek (2011) highlighted in their review, body size influences fundamental features of cladoceran ecology, including metabolic rate, feeding and growth rates, duration of juvenile development, and reproduction potential, thereby determining competitive ability. Moreover, small and large cladoceran species have different life strategies (Hart and Bychek 2011) due to different relationships between demographic parameters. Therefore, for our experimental study, we selected representatives of two species that differ considerably in size: large *Daphnia magna* Straus and small *Daphnia longispina* O. F. Müller.

We proposed that understanding the stoichiometric linkages between species within food chains is a key knowledge required to predict competition-mediated community dynamics between small- and large-bodied species. The goal of this research was to determine how changes in algal quality (stoichiometric ratios of C:P) influenced R* (based on population growth rate) and the outcome of competition between two *Daphnia* species which differed significantly in their body sizes. Based on GRH, we expected large *Daphnia* to be more sensitive to P limitation than small *Daphnia* due to their higher P demand. Consequently, we hypothesized that under high-food-quality conditions (i.e., high P-content, or low C:P ratio in the algae), large-bodied species will have lower R*, whereas under P-limiting conditions (i.e., low P-content, or high C:P ratio in algae), the small-bodied species will be superior because it requires less P for growth. Thus, we proposed that changes in the P content of algal resources will cause a shift in dominance between small- and large-bodied cladocerans. We conducted a combination of competition, life table and computer experiments to determine how algal food quality, based on variations in P content, impacted R* and competitive interactions between large- and small-bodied *Daphnia*. The computer model was developed to predict outcomes of species competition under different combinations of food supply and food quality scenarios. Competition experiments supplemented by life table experiments and computer simulations allowed us to make more general conclusions about competitive interactions between small- and large-bodied species. In addition, we regarded that competition and life table experiments conducted in the similar conditions will verify Tilman’s resource competition theory (Tilman 1981, 1982) for species with complex age structure. The knowledge about the mechanisms that facilitate shifts in competitive superiority based on food conditions will help us understand better species structure in freshwater lakes.

## Methods

### Laboratory experiments

We performed competition experiments with clones of large-bodied *D. magna* (clone DMN—from Novy Vrbensky Rybnik, Czech Republic) and small-bodied *D. longispina* (clone GB01 – from Grosser Binnensee, Germany). The clones are maintained in stock cultures of the Department of Hydrobiology, University of Warsaw. Individual size (body length) at first reproduction in the *D. magna* clone (*N* = 18) was almost twice that of *D. longispina* (*N* = 18), while body mass was almost five times greater (*N* = 18 for both species) (Table S1), and these differences far exceed the variation between clones within each of the species. If large differences in body size did not affect the competitive ability of the two genotypes, this would imply that factors other than body size play a decisive role in the competition between the cladocerans.

In the competition experiments, daphnids were kept in glass jars filled with 400 mL of experimental medium which was prepared as a 1:2 dilution of filtered lake water with demineralized water. Lake water was filtered through a Sartoban® capsule filter (pore size 0.2 µm) to remove microorganisms, including bacteria. We diluted lake water to reduce the concentrations of P in the medium.

We manipulated the amount of P in the media to create two algal treatments that differed in P content. We used the green alga *Chlamydomonas klinobasis* (strain #56, Limnological Institute, University of Constanz). *C. klinobasis* was grown in batch cultures (0.5 L Erlenmayer flask filled with 0.2 L of medium), either in full WC medium (P-rich medium), or in WC medium depleted with phosphorus (P-poor medium, 0.1 × of nominal P- content). The C content was measured by the dry combustion method using a Flash 2000 Elemental Analyzer (Thermo Scientific™, USA). The P content was measured using a continuous flow San + + Skalar analyzer (molybdate method) and the microwave-assisted sample digestion (ultraWAVE apparature) in mixture of nitric acid and peryhydrol. The C content of the algal suspensions was determined by reference to photometric light extinction at 800 nm using Perkin-Elmer spectrophotometer (Perkin-Elmer, USA), along with carbon extinction equations determined previously. The final C:P ratio in algae from P-poor medium was ~ 800 (Low P, LP), whereas in algae from P-rich medium, it was ~ 100 (High P, HP). Harvested algae were stored in the fridge at 8 °C until use.

*D. magna* and *D. longispina* were grown separately (monocultures of each species) and together (mixed culture of both species) in the experiment at 24 °C. Neonates to start the experiment were obtained from mothers that had been kept in the same medium which was used in the experimental treatments, i.e., containing algae with HP or LP contents. We initially put 5 neonates (12 h old) of *D. magna*, and 20 neonates of *D. longispina* in 400 mL jars. We used a higher initial number of newborn individuals of *D. longispina* in order to balance the biomasses and total filtering rate of each species in the inoculum (based on data reviewed by Lampert 1987). Mono- and mixed cultures of daphnids were fed with P-rich *C. klinobasis* (C:P ≈ 100) and P-poor *C. klinobasis* (C:P ≈ 800) twice a day with a 12 h interval in the amount that corresponded to 0.09 mgC L^−1^. In total, we had 6 treatments including each species in monocultures and mixed cultures at both food types (LP and HP). All the treatments were replicated in triplicate, resulting in a total of 18 jars.

Each day, all animals were transferred with a pipette to clean, autoclave-sterilized jars containing fresh medium. Every 6th day, we counted all the individuals in each jar distinguishing between neonate and adult individuals. On the day before counting, we measured chlorophyll concentration just before the evening feeding in each jar (PhytoPam fluorometer, Heinz Waltz GmbH, Germany). Animals were subjected to a summer photoperiod (16L:8D) with 10.3 µmol m^−2^ s^−1^ light intensity (Li-Cor 189 quantum sensor, Li-Cor Biosciences®, USA). The jars were placed in a water bath at 24 °C that was maintained constant with ± 0.1 °C accuracy using Zefir (Adarex, Poland) submersible thermostats. Experiments lasted 60 days during which a minimum of chlorophyll concentration was achieved for 10 days and further was kept at relatively similar low level that created conditions for competitive interactions.

Life table experiments with *D. longispina* and *D. magna* were conducted to assess R* based on the population growth rate (*r*). The experiments were performed with the same clones of *D. magna* and *D. longispina* in the medium described above at 24 °C. One female with eggs was put in a 100 mL glass. *Daphnia* spp. were transferred to clean, sterilized jars with fresh medium every day. Animals were given two types of food quality twice a day with P-rich *C. klinobasis* (C:P≈100, HP treatment) and P-poor *C. klino*basis (C:P≈800, LP treatment) at a 12 h interval. We conducted the life table experiments using three food concentrations which were required to assess R* at each food type: 0.27, 0.18, and 0.09 mg C L^−1^. We kept one female with eggs in each of the 10 replicates for each concentration and each food type until neonates were released. When neonates appeared, we left only one randomly chosen individual in the glass. The other individuals and females were removed. Their development was monitored daily to record the age of first reproduction and the number of offspring. The experiment lasted until hatching of neonates from the third clutch as later reproduction has been shown to have only a minor effect on *r* (Tessier and Woodruff 2002). After the neonates were counted, they were removed from glasses. For each species and each treatment, 10 replicates (glasses) were initially established, which in some treatments were reduced to a minimum of 6 later because some animals were lost during experiment manipulations.

Population growth rate (*r*) was estimated with Euler–Lotka equation (Kot 2001; Fujiwara and Diaz-Lopez 2017):

$$1 = \sum {l}_{x}{m}_{x}{e}^{-rx}$$

where l_x_ is age specific survival, m_x_ is age specific fecundity, and x is age in days. The sum is taken over the duration of the experiment or from x = 1 until the day of the third clutch.

We used the regression relationships between population growth rates (*r*) (y axis) and food concentrations (x-axis) where the intercept of the regression line at the x-axis was accepted as R*, i.e., that was the food concentrations at which *r* was zero. We determined R* of each species grown in HP and LP treatments. The species with the lowest population R* was regarded as the superior competitor at a given food quality.

We measured C:P in 3-day-old juveniles and adults of each *Daphnia* species. We reared individuals in the favorable conditions fed with HP *C. klinobasis* in the amount of 1 mg C L^−1^. Before sampling, we kept them in the filtered medium (free of food particles) for 6 h to empty their guts. They were then transferred to tin boats with approximately 20–30 mg of wet weight biomass per boat for each age group of each species separately. A total of 4 replicates per group were dried at 60 °C for 24 h. After cooling in the exicator, they were subjected to stoichiometric analysis. C content was measured by the dry combustion method using a Flash 2000 Elemental Analyzer. P content was measured using a continuous flow San + + Skalar analyzer (molybdate method) and the microwave-assisted sample digestion (ultraWAVE apparature) in a mixture of nitric acid and peryhydrol.

### Computer modeling

We predicted population dynamics in mono- and mixed cultures at different food supply using the escalator boxcar train (EBT) model approach (De Roos et al. 1992; De Roos 1997; Rinke and Vijverberg 2005). This framework allows us to divide a population into distinct age classes that are characterized by individual properties (e.g., by specific relationships of somatic growth rate, fecundity, birth and death rates and ingestion rate with food concentration), thus creating a stage-structured population model. The algorithm of calculation was written in C + + programming language and predicted the population dynamics at different scenarios of food supply and food quality. The species in the model were described by a set of relationships between each of the demographic parameters (individual growth rate, fecundity, mortality, ingestion rate) and food concentration at HP and LP food conditions.

At each 3-h time step of the model, we calculated food concentration:

$$C\left( {t + 1} \right) = C\left( t \right) + P \times C\left( t \right) {-} k \times C^{2} \left( t \right) {-} \sum {IR\left( {C,S} \right) \times N\left( {t, S} \right)}$$

where *C (t)* is food concentration at time *t*; *P* is daily production coefficient; *k* is coefficient of the equation of logistic growth of food concentration which determines the maximum potential food concentration; *S* is stage of the species. We distinguished 3 stages that included two premature and one adult stage. We did not include more stages because we regarded that the greatest differences in parameters existed between newborns, juveniles and adults. Ʃ*IR* – in monocultures, it is a sum of ingestion rates of the individuals of three stages while in the mixed cultures, it is a sum of ingestion rates of the individuals of three stages of both species; *N* is the number of individuals of particular stage at time *t.* We simulated two levels of food supply. Maximum food concentration which algae could reach during logistic growth at low food supply was 0.09 mg C L^−1^ and at high food supply, it was 0.54 mg C L^−1^.

At every step of the model, death rates at each stage and the number of newborns were calculated according to their relationships with food concentration at particular time (*t—τ*) where *τ* was a three-day delay because we supposed that population parameters did not respond immediately but with a delay. Thus, the number of individuals at each step was found as follows:

$$N\left( {t + 1} \right) = N\left( t \right){-}DR\left( C \right) \times N\left( {t, S} \right)$$

$${N}_{n}={N}_{a}(t)\frac{F(C)}{{D}_{e}}$$

where *N* is the number of individuals of particular stage at time *t*; *S* is a stage of development (two juvenile stages and one stage of adults); *N*_*a*_ is the number of adults at time *t*; *N*_*n*_ is the number of neonates; *DR* is death rate as a function of food concentration *C*; *F* is the number of eggs per females divided by *D*_*e*_ which is egg development time.

Functions of individual demographic parameters including death rate, fecundity and duration of development until maturity or age at first reproduction on the food concentration were described in the separate blocks of the program which the model address to at every time step. We found relationships between each demographic parameter and food concentration approximated by linear functions for HP and LP conditions in the life table experiments. The values of ingestion rate were based on the established range of filtering rates in cladocerans (Suschchenya 1975; Porter et al. 1982; Romanovsky and Feniova 1985) (see Sheet 1 *Model Verification* in Supplementary Materials). R* of the modeled *Daphnia* species was calculated based on population growth rates (*r*) of the modeled species in the similar way as described above for experimental *Daphnia*.

### Statistical analysis

We used two-way repeated measures ANOVA to test the effect of species identity (*D. longispina* vs. *D. magna*) and algal food quality (low vs. high C: P ratios) on the density of daphnids reared in mono- and mixed culture, as well as on the chlorophyll concentrations in the experimental jars. In order to eliminate the effects of arbitrarily set of initial population densities of both *Daphnia* species on their subsequent dynamics, data from the first two weeks after the start of the experiment were excluded from the analysis.

We used two-way analyses of variance (ANOVA) to test the effects of species identity (*D. longispina* vs. *D. magna*) and age class (neonate vs. adult) on C:P ratios in bodies of *Daphnia*. We used 4 replicates for each species and age (3-day old individuals and adults) which were independent of each other.

We performed residual analysis to assess deviation of the model data from the experimental data. To test the normality of the distribution of residuals, we used Shapiro–Wilk test (SW test). This test is based on comparison of the quintiles of the fitted normal distribution to the quintiles of the residuals. The *P* value of the SW test was over 0.05. Hence, the hypothesis that the residuals were distributed normally with 95% confidence was not rejected. In addition, we used correlation and regression analyses which showed that the computer model predicted abundance dynamics with high accuracy (the coefficient of determination was 0.75; Pearson correlation coefficient was 0.86). All the statistical parameters of the model verification are given in Supplementary material for one treatment (see Sheet 2 *Model Verification* in Supplementary Materials).

Tukey’s post hoc test (*P* < 0.05) was used to determine which treatments differed when significant treatment effects or interactions were detected with RM-ANOVA. Statistical analysis was performed using Statistix 10 software (Analytical Software, FL USA).

## Results

### Laboratory experiments

**Fig. 1:**
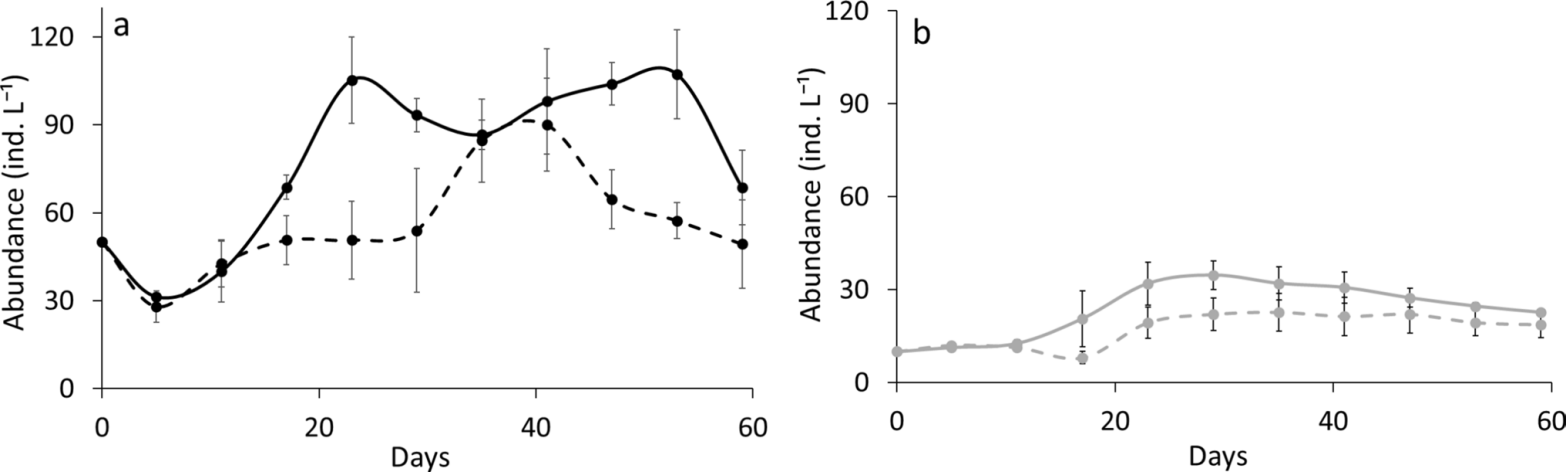
Population abundances (means ± SD) of *D. longispina* (**a**) and *D. magna* (**b**) under high phosphorus (HP, solid line) and low phosphorus (LP, dashed line) conditions in the monocultures

The two *Daphnia* species responded differently to algal C:P ratios in monocultures (RM-ANOVA, interaction species × food type: df = 1, F = 7.69, *P* < 0.01) (Fig. 1). *D. longispina* was significantly less abundant when subjected to P-poor diet compared to P-rich conditions (Tukey HSD, *P* < 0.05), whereas abundance of *D. magna* did not differ between LP and HP treatments (Tukey HSD *P* > 0.05) (Fig. 1).

**Fig. 2:**
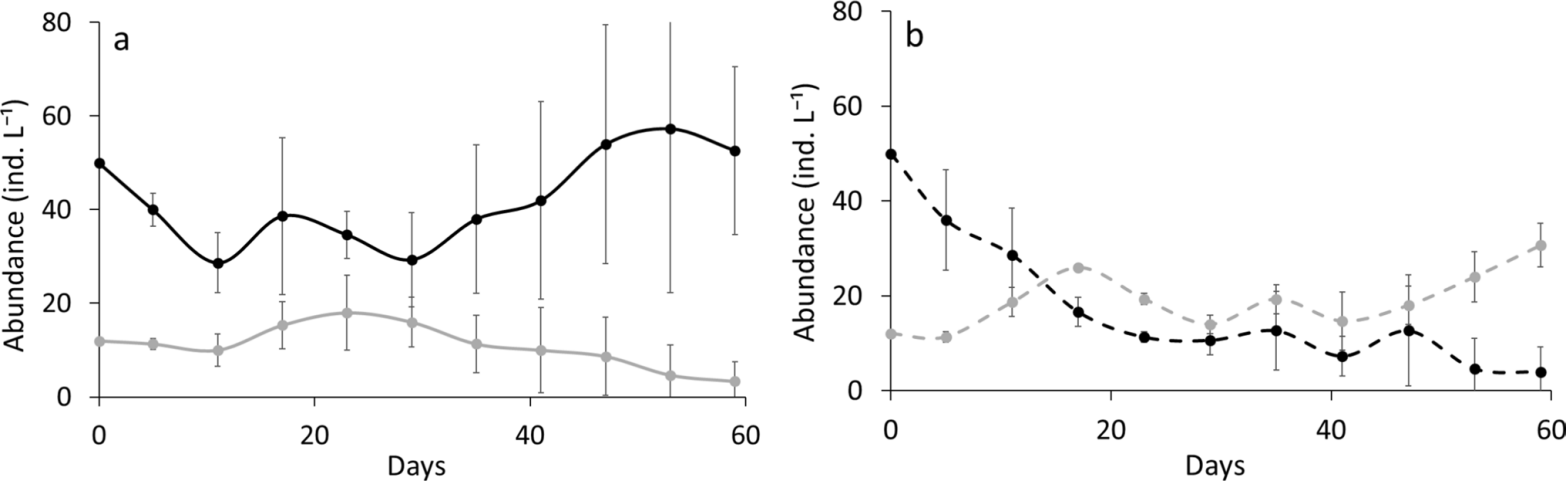
Population abundances (means ± SD) of *D. longispina* (black line) and *D. magna* (gray line) under HP (**a**) and LP (**b**) conditions in the mixed cultures

The outcome of competition experiments differed between LP and HP conditions (Fig. 2). Contrary to our expectation, *D. longispina* dominated under P-rich food conditions (Fig. 2a), while *D. magna* was superior under P-poor food conditions (Fig. 2b). Abundance of *D. longispina* and *D. magna* in the mixed cultures differed from each other and competitive superiority depended on the food type (RM-ANOVA interaction species × food type: df = 1, F = 57.3, *P* < 0.0001) (Fig. 2).

**Fig. 3:**
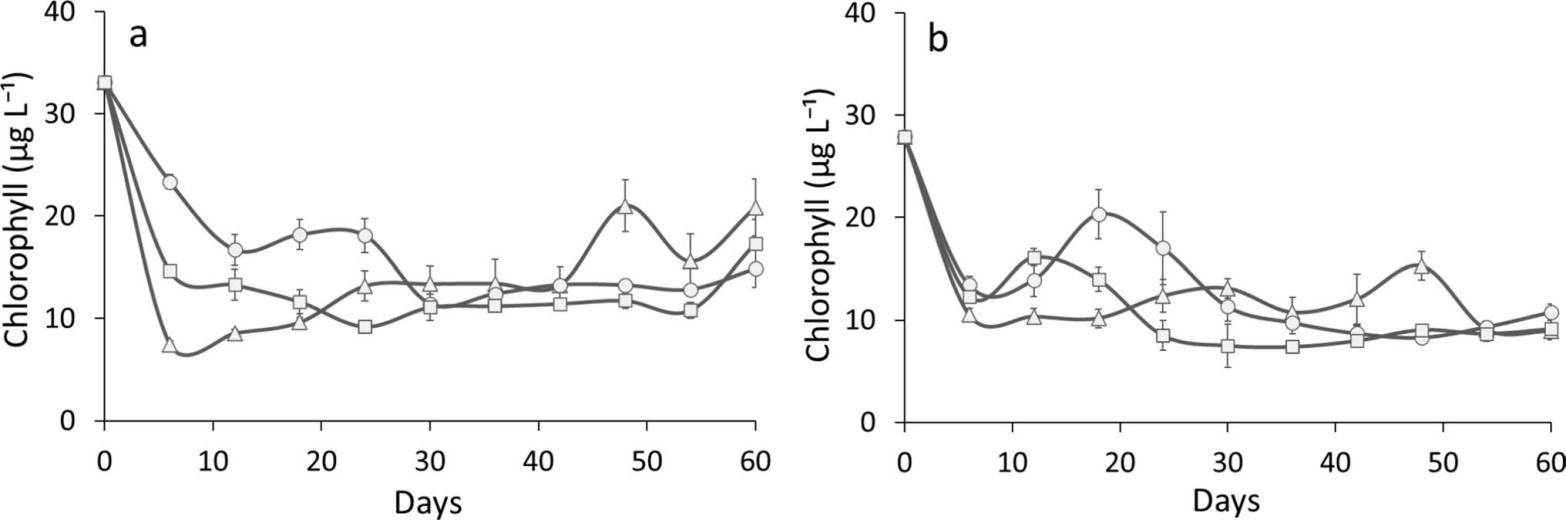
Concentration (means ± SD) of chlorophyll under HP (**a**) and LP (**b**) conditions in the monocultures of *D. longispina* (circle) and *D. magna* (triangle) and in the mixed treatment (square)

In all the monoculture treatments with *D. longispina*, chlorophyll (taken as a measure of algal abundance) was higher than in the treatments with *D. magna* or mixed cultures (Tukey HSD *P* < 0.05) (Fig. 3, Table S2). Concentrations of chlorophyll in the treatments with *D. magna* and the mixed cultures did not differ from each other (Tukey HSD *P* > 0.05). The higher concentrations of chlorophyll were observed in the treatments with *D. longispina* and in the mixed cultures of both species in the HP conditions relative to the LP treatments (Tukey HSD *P* < 0.05). There was no difference in chlorophyll concentration between mono- and mixed cultures of both *Daphnia* in the LP food conditions (Tukey HSD *P* > 0.05).

**Fig. 4:**
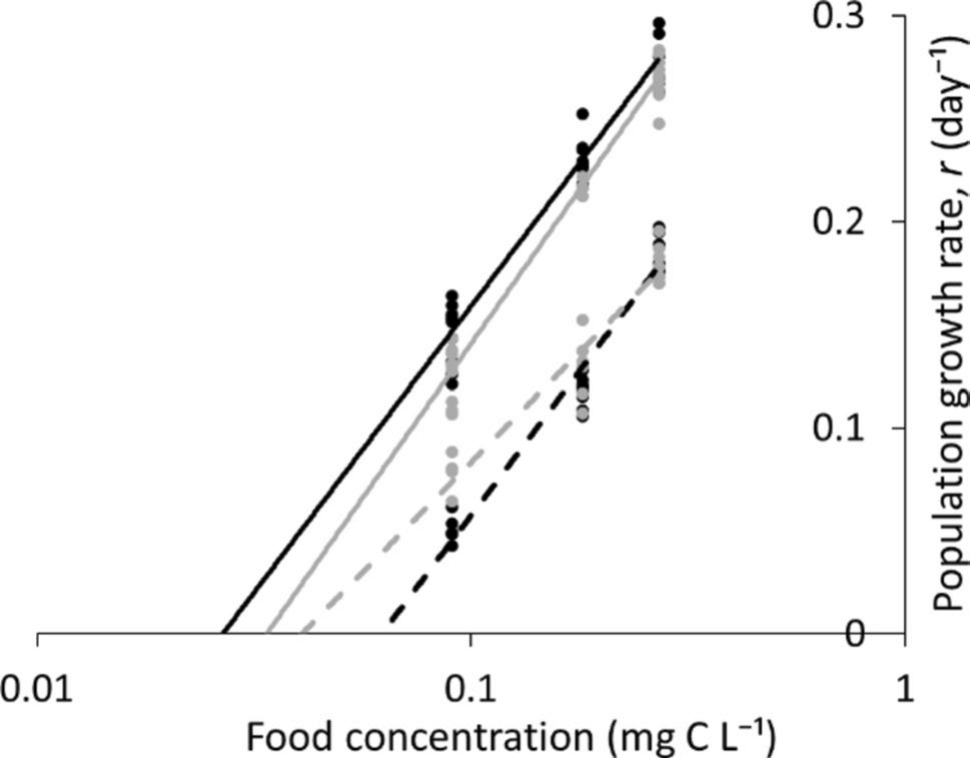
Regression relationships between population growth rate (*r* day^−1^) of the species and food concentration under HP (solid lines) and LP (dashed lines) conditions. *D. longispina –* black, *D. magna –* gray (laboratory experiment). R* was determined in the point where the regression line crossed the x-axis. Note log scale used in panel

Regression relationships between population growth rates (*r*) of the species and food concentration showed that at HP, R* of both species was lower than the respective values at LP (Fig. 4, Table S3). At HP, R* of *D. longispina* was lower than that of *D. magna*, while at LP, R* of *D. magna*, on the contrary, was lower.

**Fig. 5:**
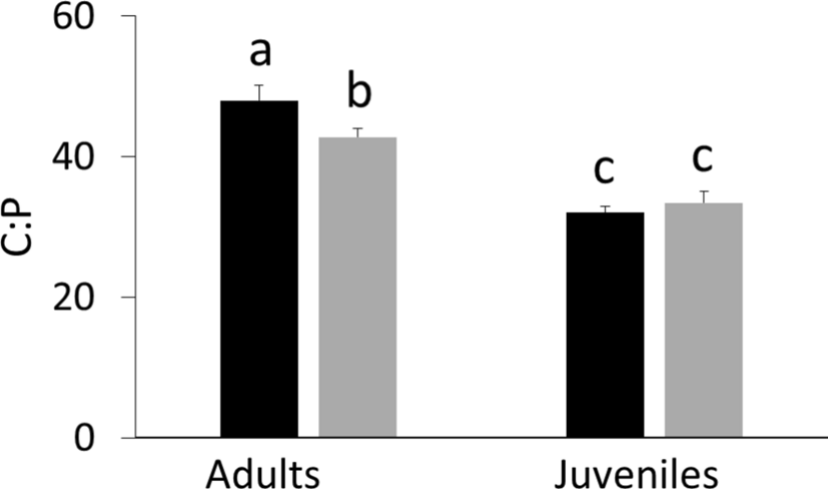
C:P ratios (means ± SD) in adults and 3-day old juveniles of *D. longispina* (black) and *D. magna* (gray). Different letters indicate significant differences based on* P*-values from Tukey’s post hoc tests

Adult *Daphnia* had significantly higher C:P ratios than three-day-old juveniles in both species (Tukey HSD *P* < 0.001 for adult vs. juvenile) (Fig. 5). The C:P ratios also differed between adults of the two species, but not between the juveniles as indicated by the significant age × species interaction (f = 1, F = 16.1, *P* = 0.001). Adults of *D. longispina* (48.0 ± 1.08; mean ± SD) had significantly (Tukey HSD, *P* < 0.001) higher C:P ratios than adults of *D. magna* (42.8 ± 0.64) (Fig. 5).

### Computer competition experiments

**Fig. 6:**
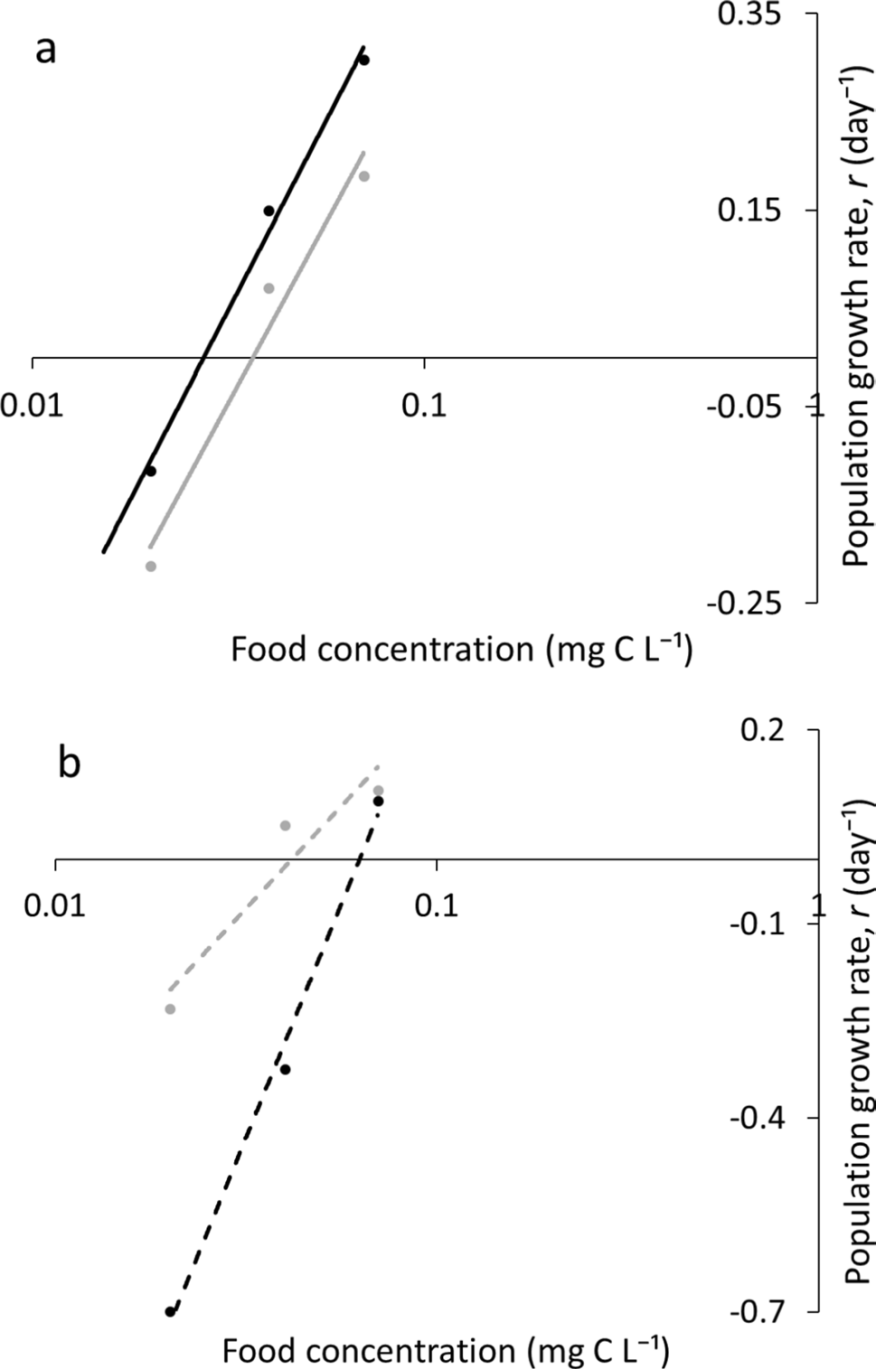
Regression relationships between population growth rate (*r* day^−1^) of the species and food concentration under HP (solid lines, **a**) and LP (dashed lines, **b**) conditions. Modeled *D. longispina –* black, modeled *D. magna –*gray (computer simulation). R* was determined in the point where the regression line crossed the x-axis. Note log scale used in panel

R* of the modeled species was close to R* obtained in the life table experiments (Fig. 6, Tables S3 and S4). R* at HP was lower than the respective values at LP for both species. However, at HP, the modeled *D. longispina* had lower TFC than the modeled *D. magna*, while at LP, the opposite pattern was observed.

**Fig. 7:**
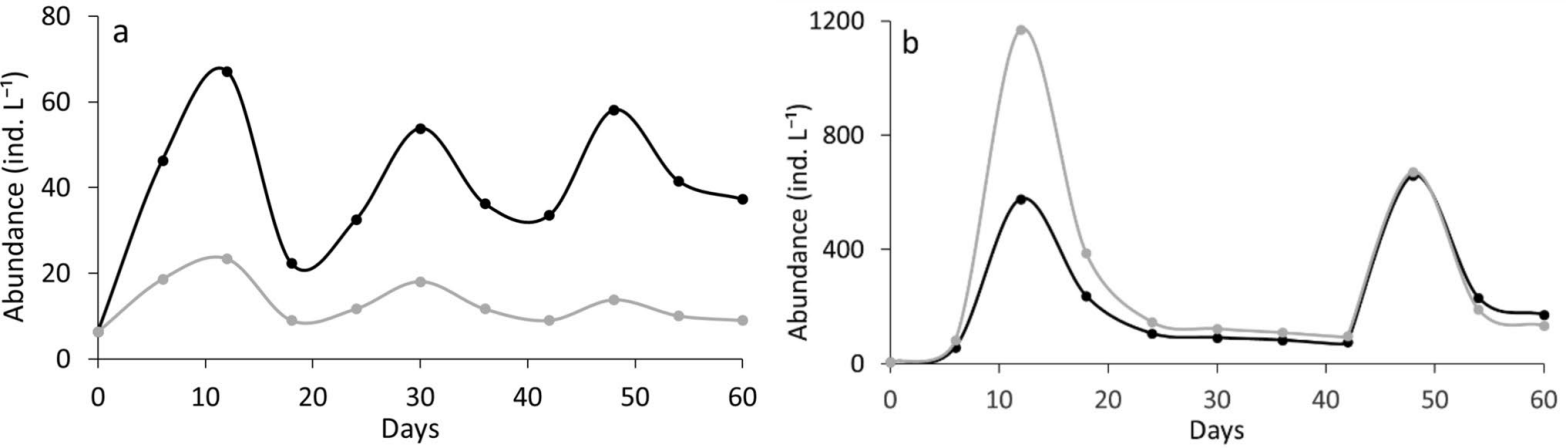
Population dynamics of modeled *D. longispina* (black) and *D. magna* (gray) under HP conditions in the mixed cultures for the cases of low food supply (**a**) and high food supply (**b**)

Under HP conditions, the modeled *D. longispina* was more abundant than modeled *D. magna* in the computer competition experiment with low food supply (Fig. 7a). However, in high food supply experiment, the modeled *D. magna* dominated during the first peak and abundance of both species was similar during the second peak. (Fig. 7b).

**Fig. 8:**
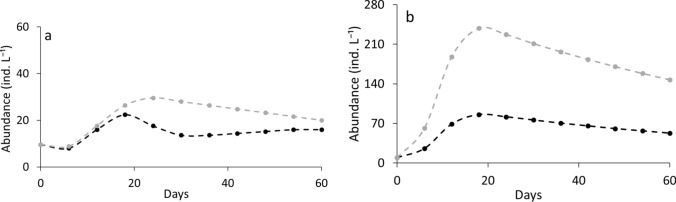
Population dynamics of modeled *D. longispina* (black) and *D. magna* (gray) under LP conditions in the mixed cultures for the cases of low food supply (**a**) and high food supply (**b**)

Under LP conditions, at low food supply, the modeled *D. magna* dominated which was in accordance with its lower R* (Fig. 8a). At high food supply (Fig. 8b), the abundance of the modeled *D. magna* exceeded that of the modeled *D. longispina*.

## Discussion

We hypothesized that at high P content or low C:P ratio in the algae, large-bodied species would be superior. In contrast, under low P content or high C:P ratio in the algae, large-bodied species should be inferior. Our assumptions were based on the fact that P is a key dietary element for many physiological processes, especially for somatic growth since P is mainly associated with ribosomal RNA (Elser et al. 1996, 2003; Loladze and Elser 2011). In support, we found that R* of both species increased under P limitation relative to HP conditions. As a result, *Daphnia* abundance was higher in HP than in LP treatments in monocultures. This finding is in accordance with compensatory feeding theory (Boersma and Kreutzer 2002; Iwabuchi and Urabe 2012b). Specifically, under P limitation conditions, animals consume more carbon to meet the requirements for P.

We initially expected that large-bodied species would have lower C:P ratios as was indicated in some previous studies (Andersen and Hessen 1991; Bergström et al. 2015). However, the P content was similar in juveniles of both *Daphnia* species, while adults of the small-bodied *D. longispina* had lower P content than its large-bodied competitor. This measurement cannot explain why large *D. magna* did better at P-limiting conditions. However, the same paradoxical situation was described by Iwabuchi and Urabe (2010). They found that P contents of the small-bodied *Ceriodaphnia quadrangula* (O.F. Müller, 1785) and large-bodied *Daphnia pulex* Leydig, 1860 were similar. However, *D. pulex* grew faster at P-limiting conditions. They proposed the following reasons to explain this phenomenon: *C. quadrangula* acquired P less efficiently, expended a higher fraction of assimilated P on maintenance, or both. In addition, the largest loss of P occurred through molting (He and Wang 2007). Since small-bodied cladocerans have a higher surface-to-volume ratio, they lose a larger fraction of P through molting than large-bodied competitors, which is reflected in growth under P-limiting conditions. These results indicate that body P content cannot explain the difference in growth rates of the two species.

Likely due to the above reasons, small-bodied *D. longispina* was more sensitive to P limitation. Indeed, we found that the magnitude of the rise of R* in *D. longispina* was greater than that of *D. magna* under P limitation relative to HP conditions*.* Specifically, *D. longispina* fed with LP algae had a higher R* than *D. magna*, whereas R* of *D. longispina* fed with HP diet was lower. This indicated that if we regard R* as a predictor of competitive superiority (Tilman 1981, 1982), *D. longispina* will suppress *D. magna* at HP conditions while *D. magna* will be superior at LP.

Results of our competition experiments under LP and HP were in line with the predictions based on R*: the dominant species had a lower R*. In particular, *D. longispina* suppressed *D. magna* at HP conditions, while *D. magna* was superior at LP food conditions. Thus, we found a shift of superiority between the species in competitive experiments in accordance with R*. Since R* changed in response to resource C:P, we can conclude that the P-content of algae controls the outcome of competition. In addition, our results suggest that Tilman’s resource competition theory is justified for animals with complex age structure as cladocerans. Noteworthy is that Tilman’s theory was previously verified only for diatoms and rotifers, i.e., species with no distinguished differences between age classes (Tilman 1981; Rothhaupt 1988).

Iwabuchi and Urabe (2012a) were the first who showed that competitive interactions between *Daphnia* species depended on C:P ratio in algal food. Our results are in agreement with this finding. However, these authors did not reveal shift in superiority in dependence on algal P-content. They established only the changes in magnitude of suppression of one species by the other in dependence of algal P content which was assessed as a ratio of abundance in mixed culture to that in monoculture. The reason for this could be that they conducted relatively short-term experiments which lasted only 25 days (two generations) while our experiments lasted 60 days (about 6 generation). Maternal effects could mask the effect of P in their short-term experiments. The other difference between our studies was the assessment of TFC: Iwabuchi and Urabe (2012b) assessed TFC based only on the somatic growth of juvenile individuals, while we determined R* (population TFC) in the life table experiments. We believe that R* is a more accurate index of competitive ability than TFC found based on juvenile weight because R* measurements involve the values of the whole set of the demographic parameters including fecundity, duration of development until maturity, etc. Furthermore, Iwabuchi and Urabe assessed somatic growth TFC for a fixed interval of time (5 days). In this regard, Lampert and Trubetskova (1996) showed that somatic growth rate is a good predictor of population growth rate only if it is measured for the whole period of juvenile development until the first clutch because in this case the final weight included reproductive material. Moreover, weight is a highly variable parameter and for this reason in Iwabuchi and Urabe (2012b), 95% confidence interval of TFC for the studied species was large which could mask the effects of algal P-content on TFC.

We believe that we have made an important step in understanding competitive interactions showing that even small differences in R* can result in shifts in the superiority between *Daphnia* species that differ considerably in body size. Noteworthy is that we created conditions where the species competed for one resource. Iwabuchi and Urabe (2010) showed that bacteria play a crucial role in the outcome of competition between cladocerans especially if one of the competitors is more favored by bacteria than its counterpart. To minimize the development of bacteria, we transferred the *Daphnia* daily to sterilized jars with fresh media. If additional food sources were present, including bacteria, R* would not be a good predictor of competitive ability, as there may be a separation in the food spectrum between *Daphnia* species. Additionally, we found that R* was lower in small-bodied species, yet the size gives considerable advantage when the food supply is high.

The large-bodied species *D. magna* has a higher reproduction potential relative to small-bodied species due at least in part to a larger brood chamber, which enables them to produce more offsprings (Bartosiewicz et al. 2015; Luhring et al. 2018). However, this advantage can be only realized at high food concentrations, when there is no carbon limitation. In support, our computer experiments revealed that in cases when food supply was increased three-fold, competitive advantage in production of a higher number of newborns during even short periods of high food concentrations gave the advantage to *D. magna* allowing it to dominate at HP, while at low food supply, this species was inferior. Thus, high food supply can facilitate large *Daphnia* to quickly occupy the space and suppress competitors due to high abundance. For this reason, large *Daphnia* in nature tends to demonstrate an *r*-strategy and successfully inhabits eutrophic waterbodies (Romanovsky 1984).

Hence, our results suggest that Tilman’s resource competition theory was justified for cladoceran populations with distinguished age structure only when food supply (analog of primary production) in the experiment was low. When food supply was high, the species with higher reproduction potential will be superior. This result is in accordance with the conclusions of Tessier et al. (2000) stating that when food resource was abundant, the species with the highest maximum growth rate was favored, while at poor resource conditions, the species with lower minimum resource requirements was superior. In this regard, Tessier et al. (2000) indicated that there is a trade-off between the ability to rapidly exploit rich food conditions vs. the ability to use poor food environment. These features are adaptive and influence the distribution of the species or clones with particular traits among lakes consistently (Tessier and Woodruff 2002).

Shifts in superiority between competing large- and small-bodied cladoceran species have been observed in nature. For example, cladoceran communities in rock pools studied on small Baltic islands consisted of only three *Daphnia* species (the smallest species *D. longispina*, medium-sized* D. pulex* and the largest species *D. magna*) which competed for food and had no predators (Ranta 1979). Regardless of similar competitive conditions, there were great varieties of the species structure in the pools. Hanski and Ranta (1983) explained different competitive outcomes between three *Daphnia* species by their differences in competitive advantages. Specifically, they stated that *D. longispina* was the best competitor, while *D. magna* was the best colonizer (fast spread). Due to its advantage in high reproductive potential and fast spread, *D. magna* was able to colonize the pool very fast after it dried and then filled with water, which was called a “priority effect”. Our experimental results are in accordance with these observations. Computer models showed that production of greater number of neonates by *D. magna* at the beginning of the experiments at high food supply made this species dominate likely due to “priority superiority”. Bengtsson (1987) also indicated that large *D. magna* was the best competitor at higher food supply, while *D. longispina* was dominant at lower food levels. In accordance, Romanovsky and Feniova (1985) indicated that small-bodied cladocerans outcompeted large species when food supply is scarce, but they were outcompeted at higher food supply.

We propose that fluctuating food quality may cause shifts in the superiority of competing species. This may allow them to coexist. Specifically, *D. magna* will dominate at high primary production and any C:P ratio in the seston, or at low primary production but high C:P. However, *D. longispina* has a chance to be competitively superior at low primary production and low algal C:P. We should point out that we do not claim that we completely understand the competitive interactions between *D. longispina* and *D. magna* because we used single clones of each species. There may be crucial differences between clones which could alter our expectations. Furthermore, we used only one pair of competing species. We think that the main point of this study is to show that food P content may affect R* which, in turn, can help determine the outcome of competition, yet, only at low food supply.

We concluded that there may be shifts in competitive superiority between *Daphnia* species depending on the elemental content of food and the level of food supply. *D. magna* was a superior competitor at low P content while *D. longispina* dominated at high P content. We also found that enhanced food supply gave an additional advantage to the large-bodied *D. magna* due to higher reproduction potential at high food concentrations.

## Supplementary Information

Below is the link to the electronic supplementary material.

Supplementary file1 (XLS 88 kb)

## Data Availability

Data will be made available by the authors upon reasonable request.
